# Acceptance of criteria for health and driver scoring in the general public in Germany

**DOI:** 10.1371/journal.pone.0250224

**Published:** 2021-04-22

**Authors:** Felix G. Rebitschek, Gerd Gigerenzer, Ariane Keitel, Sarah Sommer, Christian Groß, Gert G. Wagner

**Affiliations:** 1 Harding Center for Risk Literacy, Faculty of Health Sciences Brandenburg, Potsdam, Germany; 2 Max Planck Institute for Human Development, Berlin, Germany; 3 Federal Ministry of Justice and Consumer Protection, Berlin, Germany; 4 Advisory Council for Consumer Affairs, Federal Ministry of Justice and Consumer Protection, Berlin, Germany; 5 Alexander von Humboldt Institute for Internet and Society (HIIG), Berlin, Germany; 6 German Socio-Economic Panel Study (SOEP), Berlin, Germany; Universitat de Valencia, SPAIN

## Abstract

Numerous health insurers offer bonus programmes that score customers’ health behaviour, and car insurers offer telematics tariffs that score driving behaviour. In many countries, however, only a minority of customers participate in these programmes. In a population-representative survey of private households in Germany (N = 2,215), we study the acceptance of the criteria (features) on which the scoring programmes are based: the features for driver scoring (speed, texting while driving, time of driving, area of driving, accelerating and braking behaviour, respectively) and for health scoring (walking distance per day, sleeping hours per night, alcohol consumption, weight, participation in recommended cancer screenings, smoking status). In a second step, we model participants’ acceptance of both programmes with regard to the underlying feature acceptance. We find that insurers in Germany rarely use the features which the participants consider to be the most relevant and justifiable, that is, smoking status for health scoring and smartphone use for driver scoring. Heuristic models (fast-and-frugal trees) show that programme acceptance depends on the acceptance of a few features. These models can help to understand customers’ preferences and to design scoring programmes that are based on scientific evidence regarding behaviours and factors associated with good health and safe driving and are thus more likely to be accepted.

## Introduction

Healthcare prevention programmes that focus on the promotion of a healthy lifestyle and of physical activity can reduce cardiovascular events and all-cause mortality [1]. For that reason, many health care institutions consider behavioural change to be crucial, and to this end measure, monitor, and incentivise behavioural changes financially (behaviour-based tariffs). For instance, health insurers reward certain health-related behaviours with insurance premium discounts [2]. Other insurers use big data analytics to score drivers’ and other customers’ behaviours [3].

Scoring models are algorithms that process criteria of an individual (e.g. his or her characteristics, behaviour, or living conditions) to derive a value (e.g. a prediction or a classification). Scoring models can have two aims: to predict and to steer individuals’ behaviour [4]. The scoring of humans and their behaviour is traditionally found in schools, where pupils receive marks for their performances in tests, and in everyday life in the form of credit scoring. But scoring has since become a more general principle. Today, it is applied in economy (e.g. for consumer scoring) [4] and, in part, in policymaking (e.g. Chinese social scoring system developments [5]) and public service, such as predictive policing [6]). Steering behaviour of individuals via scoring has, thus, has become a rationale not only for the Chinese citizen scoring systems [5], but also for commercial systems in the Western world [7].

Incentive programmes in health insurance and telematics programmes in automobile liability insurance reward (and occasionally punish) behaviours by utilising higher and lower insurance premiums and discounts. Insurers have manifold intentions when providing scoring programmes: e.g. promoting healthy behaviour and thereby potentially reducing expenditures for preventable and chronic disease management [8] and promoting safe driving of customers to prevent costly consequences of accidents [9]. Scoring programmes are also viewed as essential marketing tools [8, 10, 11] that could give insurers a competitive edge and help them attract the attention of low-risk customers [12], such as of young people for health insurances.

Nevertheless, it remains open whether such programmes are effective in terms of their announced goals of fostering safe driving and healthy behaviour (see, e.g. the difficulties in driver scoring field research [20]. For instance, monetary rewards can support smoking cessation, vaccination and screening participation over the short term [13], but not consistently for people over 50 years of age [14]. Some authors believe health scoring has been proven to be effective and economically useful [11], even though evidence is mixed [15–17]. Those individuals who decide to participate in health scoring also more likely decide—and potentially would have decided anyway—to participate in preventive measures regarding nutrition, exercise and relaxation [18], health expenditures can increase [8]. In summary, health scoring programmes are in place, although substantial evidence on the effectiveness of implemented health and driver scoring has yet to be provided, especially randomised controlled field experiments [19]).

Customer figures indicate limited public acceptance of the existing types of behavioural consumer scoring. The uptake of driver scoring programmes in Germany, promising savings of up to 30% of premiums, appears to be quite limited. Only about 300,000 among 40 million licensed drivers take part in driver scoring (2019). The number of insurers who offer telematics contracts decreased from 14 (in 2017) to 10 (in 2020) [20]. The uptake of health scoring in Germany also appears to be limited: a proprietary online survey in 2017 reported that one out of four customers of public health insurers took part [21], compared with 20.4% in 2011 [22]. These observations raise a question: is the relative lack of uptake due to the criteria (features) used in the scoring programmes? In our study we test, with the help of a heuristic model, how the acceptance of scored behaviours affects the acceptance of scoring programmes.

Insurers’ pricing of driving behaviour has a long history [23], but technological developments in telematics over the last decade (GPS black boxes, the European emergency calling system eCall, telematics apps for smartphones) allow for incorporating new driving features: driving behaviour (e.g. acceleration) as well as driving conditions (e.g. night-time) which can affect premiums or discounts. Nevertheless, scoring models from today’s German car insurers for prediction of cases of damages are often less complex in terms of the number of features than research developments suggest [24, 25]; typically, the weighted average of four feature values translates into a score.

Due to the regulations governing statutory health insurers in Germany, who insure 73 out of 82 million people (German Ministry of Health 1 July 2019), incentive programmes do not influence premiums but instead provide forms of discounts. Health scoring programmes typically evaluate features such as sports activities, weight, cancer screening participation, and smoking. Although the programmes were not originally designed with telematics technology, the latter has become a component of many programmes. Apps no longer simply comprise digital booklets to document activities; some insurers now integrate wearables and step counters to score actual behaviour [26].

Very often, however, proxy values (e.g. gym membership) instead of actual behaviour (e.g. workout at the gym) determine discounts (Table 1). And insurers often fail to provide evidence [4] for why certain features are rewarded (or punished) as well as potential side effects for consumers [27]. Thus, insurers do not necessarily base their scoring models on evidence regarding the effectiveness of features in terms of an announced scoring goal.

**Table 1:** Features for driver and health scoring with their validity, and the percentage of insurers using them in Germany.

**Driver scoring**	**Validity (feature has proven effect on driving safety)**	**Percentage of insurers using feature [**4**] (N = 10)**
Exceeding speed limits	Valid [29, 30]	90%
Texting with smartphone while driving	Valid [30]	22%
Driving mostly at night	Valid [30, 31]	33% [Time: 100%]
Driving mostly in urban areas	Unknown [32, 33] (depends on type of safety event)	33% [Route type: 70%]
Accelerating or braking recklessly	Valid [34, 35] (driving with foresight)	100%
Driving long hours	Valid (e.g. exhaustion)	11%
**Health scoring**	**Validity (feature has proven positive effect on health)**	**Percentage of insurers using feature [**4**] (N = 45)**
Walking 6 km per day	Valid [36, 37]	< 5%
Sleeping 7 to 8 hours per night minimum	Unknown [38, 39] (e.g. about more than 8 hours)	0%
Limited intake of alcohol	Valid [40]	0%
Normal weight	Unknown [41] (baselines)	18%
Participating in recommended cancer screenings	Unknown [42]	100%
Being non-smoker	Valid [43]	16%
Health course participation	Valid (if confirmed activity)	91%
Gym membership	Unknown (proxy activity)	91%
Participation in sports events (e.g. marathon)	Unknown (proxy activity)	64%
Sports awards and badges	Valid (confirmed activity)	91%
Vaccination status	Valid (if recommended)	91%
Health apps & wearable purchases	Unknown (proxy)	7%

In the following we explore to what extent a specific scoring feature is evaluated by consumers as being justifiable and whether these evaluations predict the acceptance of scoring programmes. To that purpose, we propose and test a heuristic model, a transparent fast-and-frugal decision tree for classification under uncertainty [28]. Additionally, we contrast consumers’ evaluations with evidence on the actual use and effectiveness of scoring features reported by insurance companies.

## Materials and methods

Our aim was to study how a representative sample of the population in Germany evaluates features for a scoring-based pricing of health and car insurances, with a between-subjects design that assigned participants to questions about driver or health scoring, and either to a condition with bonus framing or, with regards to potential penalties or behavioural punishment, to a condition with “malus” framing. Participants evaluated five features for driver scoring (speed, texting while driving, time of driving [day vs. night], area of driving [city vs. countryside], accelerating and braking), and six features for health scoring (walking distance per day, sleeping hours per night, alcohol consumption, weight, participation in recommended cancer screenings, smoking status).

Together with the Advisory Council for Consumer Affairs—independent experts from various disciplines who advise Germany’s federal Government in consumer protection policy—we developed the survey questionnaire for a population-representative survey. The survey was structured as follows: questions on participants’ health and mobility behaviour, knowledge about credit scoring (reported elsewhere [4]), driver and health scoring (between-subjects design, 2 [driver/health scoring] x 2 [bonus/malus frame]), attitudes towards novel forms of scoring that link multiple domains of life in one score (“super-scoring”, e.g. social credit scores, also reported elsewhere [4]), attitudes towards digital technologies, control beliefs, and socio-demographic data.

The survey was conducted by the fieldwork company Infas as part of a national computer-aided (to the interviewer) telephone survey (CATI) on consumer scoring from February to April 2018. An ADM telephone sample [45] was drawn according to the dual-frame approach, with fixed and mobile telephone numbers in the distribution 70% to 30%. To allow for nonresponse and ineligibility, Infas rang 110,228 phone numbers in Germany without prior notice. Of these, 88,302 numbers were not valid, 14,327 refused and 5,384 could not be contacted, were not able to participate or did not complete the survey questions. 2,215 interviews were conducted in full (utilisation rate 10%).

### Sample population

The 2,215 participants were German-speaking residents in Germany in private households with a fixed or mobile phone connection. 1,123 men and 1,092 women aged 16 to 94 participated (M = 49.2 years of age (SD = 18.9)). Participants gave verbal consent within a standard instruction that provided obligatory information and information on request, and consent was documented by the interviewers electronically (S1 Table). A separate parental consent for participants at the age of 16 to 17 is not required in Germany.

### Survey administration

The standardised questionnaire was pre-tested with 91 participants (52% female, M = 34.8 years of age [SD = 15.1]) in a group test [44] and in 48 telephone interviews [4, 45]. The average duration of interviews was 22.5 minutes. Because the willingness to participate varied among different population groups, the sample was weighted as follows: first, design transformation weighting (household to individual level) and, second, redress weighting according to crossed population features (especially age, gender, number of household members).

### Survey questionnaire

The questionnaires presented scenarios for driver scoring (car insurance) and health scoring (health insurance) [9, 21]. The driver scoring scenario was: “Imagine that a car insurance company offers a tariff that depends on the driver’s driving behaviour. One’s driving behaviour would be recorded for this purpose, for example, with a mobile phone. Whoever participates in this tariff could, depending on their own driving behaviour, influence the amount of their insurance premium.” Then participants were asked how justified they think the following regulations are: “A lower car insurance premium is paid by those who maintain the prescribed maximum speed / do not write or read phone messages while driving / mostly drive during the day / mostly drive a car in the country / accelerate or brake carefully.” This is the formulation for bonus framing. Afterwards, participants were asked whether they would personally consider using such a car insurance tariff that takes into account features such as speed, mobile phone use, acceleration and braking behaviour, time and area of driving (Yes/No/I do not know). All of the questions (bonus and malus framing) can be found in the (S2 Table).

Similarly, a health scoring scenario informed the participants: “Imagine that a health insurance company offers a tariff that depends on the health behaviour of the insured. One’s health behaviour would be recorded for this purpose, for example, with a mobile phone. Whoever participates in this tariff could, depending on their own health behaviour, influence the amount of their insurance premium. How would you evaluate the following regulations? A higher health insurance premium is paid by those who walk less than 6 kilometres per day / sleep less than 7 to 8 hours per night / drink more than small amounts of alcohol / are overweight / do not participate in recommended cancer screening tests / are smokers.” This is the formulation for malus framing.

### Analysis

Analyses were performed by logistic regressions with the dependent variable “considering participation in a scoring programme” (Yes vs. No/I do not know) across the two scorings (driver, health) to investigate the influences of respective feature evaluations. Given our assumptions, we included (besides one exploratory analysis with age, gender, and education) bonus/malus framing and respective scoring features (S3 Table) as potential predictors (without further selection all variables are entered in a single step in the model; model fit indicated by goodness of fit).

We used the FFtree [46] package for R to develop and validate fast-and-frugal decision tree models. Fast-and-frugal trees are interpretable, lexicographic decision trees with only a few features. Each feature is followed by a branch leading either to the next feature or directly to a decision. Only the last feature branches into two possible decisions [28]. The models were determined according to the “ifan” algorithm which systematically varies and truncates the tree structure for a fixed set of features, ordered by their respective balanced accuracy (bacc) in classification, in order to choose one tree (with the highest bacc) among a set (“fan”) of trees [46].

## Results

More than one third of the representative sample considered participating in driver scoring (36.0%) and health scoring (33.8%). For an overview see Table 2. Among participants holding a driver’s license, 34.1% considered participation in driver scoring (40 million out of 69.5 million adults in Germany held a type of driver’s license in 2018 [20]). Participating in driver scoring was less likely considered by people aged 50 years or above (OR = 0.63, 95%CI [0.38, 0.88], *p* < .001). Neither gender nor education affected the consideration of participating in health scoring (S3 Table; undirected analyses of any personal survey variable to influence scoring acceptance are published in a report of the company Infas for the Ministry of Justice and Consumer Protection [45]). Respondents were less likely to consider driver scoring in the case of malus systems as opposed to bonus systems (OR = 0.57 [0.31, 0.81], *p* < .001). Notably, malus framing did not alter acceptance of health scoring (Table 2).

**Table 2:** Percentage of participants who considered participation in driver or health scoring programmes and their ratings of the features as “rather” or “definitely” justified.

	Total	Framing		Gender		Age		Education ^b^		
		Bonus	Malus	Female	Male	< 50 years	> = 50 years	Low	Moderate	High
N	1,160	563	598	583	577	565	587	802	146	197
Consideration to participate in driver scoring (to join the tariff), % [95%CI]^a^	36.0 [34.6, 37.4]	42.7 [40.6, 44.8]	29.7 [27.8, 31.6]	38.1 [36.1, 40.1]	33.8 [31.8, 35.8]	41.5 [39.4, 43.6]	31.2 [29.3, 33.1]	36.3 [34.6, 38.0]	35.3 [31.3, 39.3]	33.1 [29.7, 36.5]
Rather/definitely justified use of the following features:										
Exceeding speed limits, % [95%CI]	46.3 [43.4, 49.2]	62.3 [58.3, 66.3]	31.3 [27.6, 35.0]	44.5 [40.5, 48.5]	48.2 [44.1, 52.3]	53.9 [49.8, 58.0]	39.3 [35.3, 33.3]	45.2 [41.8, 48.6]	43.7 [35.6, 51.8]	49.1 [42.1, 56.1]
Texting with smartphone while driving, % [95%CI]	72.2 [69.6, 74.8]	65.6 [61.7, 69.5]	78.4 [75.1, 81.7]	69.9 [66.2, 73.6]	74.4 [70.8, 78.0]	75.6 [72.1, 79.1]	69.2 [65.5, 72.9]	73.4 [70.3, 76.5]	67.8 [60.2, 75.4]	69.0 [62.5, 75.5]
Driving mostly at night, % [95%CI]	9.8 [8.1, 11.5]	16.9 [13.8, 20.0]	3.1 [1.7, 4.5]	8.9 [6.6, 11.2]	10.7 [8.2, 13.2]	8.0 [5.8, 10.2]	11.6 [9.0, 14.2]	11.7 [9.5, 13.9]	5.8 [2.0, 9.6]	5.9 [2.6, 9.2]
Driving mostly in urban areas, % [95%CI]	22.4 [20.0, 24.8]	29.9 [26.1, 33.7]	15.4 [12.5, 18.3]	20.2 [16.9, 23.5]	24.7 [21.2, 28.2]	19.3 [16.0, 22.6]	25.6 [22.1, 29.2]	23.9 [20.9, 26.9]	12.3 [7.0, 17.6]	22.6 [16.8, 28.4]
Accelerating or braking recklessly, % [95%CI]	46.5 [43.6, 49.4]	44.1 [40.0, 48.2]	48.7 [44.7, 52.7]	48.5 [44.4, 52.6]	44.3 [40.2, 48.4]	47.5 [43.4, 51.6]	46.0 [42.0, 50.0]	47.3 [43.8, 50.8]	42.6 [34.6, 50.6]	43.5 [36.6, 50.4]
Consideration to participate in health scoring (to join the tariff), % [95%CI]	33.8 [32.3, 35.3]	35.6 [33.6, 37.6]	31.7 [29.7, 33.7]	32.9 [30.9, 34.9]	34.6 [32.5, 36.7]	34.6 [32.5, 36.7]	33.3 [31.2, 35.4]	32.9 [31.1, 34.7]	32.3 [28.6, 36.0]	34.9 [31.6, 38.2]
N	1,055	557	498	547	507	522	524	669	157	204
Rather/definitely justified use of the following features:										
Walking 6 km per day, % [95%CI]	17.6 [15.3, 19.9]	26.1 [22.4, 29.8]	8.1 [5.7, 10.5]	14.1 [11.2, 17.0]	21.3 [17.7, 24.9]	20.2 [16.8, 23.6]	15.2 [12.1, 18.3]	16.7 [13.9, 19.5]	20.4 [14.1, 26.7]	17.9 [12.6, 23.2]
Sleeping 7 to 8 hours per night minimum, % [95%CI]	9.2 [7.5, 10.9]	13.3 [10.5, 16.1]	4.6 [2.8, 6.4]	7.6 [5.4, 9.8]	10.8 [8.1, 13.5]	11.4 [8.7, 14.1]	7.1 [4.9, 9.3]	9.3 [7.1, 11.5]	8.0 [3.8, 12.2]	8.2 [4.4, 12.0]
Very limited intake of alcohol, % [95%CI]	39.4 [36.5, 42.3]	43.0 [38.9, 47.1]	35.3 [31.1, 39.5]	41.2 [37.1, 45.3]	37.5 [33.3, 41.7]	42.0 [37.8, 46.2]	37.1 [33.0, 41.2]	38.6 [34.9, 42.3]	45.0 [37.2, 52.8]	39.5 [32.8, 46.2]
Being of normal weight, % [95%CI]	32.4 [29.6, 35.2]	35.1 [31.1, 39.1]	29.5 [25.5, 33.5]	29.5 [25.7, 33.3]	35.6 [31.4, 39.8]	33.6 [29.5, 37.7]	31.6 [27.6, 35.6]	32.3 [28.8, 35.8]	32.0 [24.7, 39.3]	35.3 [28.7, 41.9]
Participating in recommended cancer screenings, % [95%CI]	55.2 [52.2, 58.2]	63.1 [59.1, 67.1]	46.4 [42.0, 50.8]	51.2 [47.0, 55.4]	59.5 [55.2, 63.8]	60.0 [55.8, 64.2]	50.9 [46.6, 55.2]	52.2 [48.4, 56.0]	62.2 [54.6, 69.8]	60.6 [53.9, 67.3]
Being non-smoker, % [95%CI]	58.1 [55.1, 61.1]	58.5 [54.4, 62.6]	57.6 [53.3, 61.9]	58.1 [54.0, 62.2]	58.0 [53.7, 62.3]	58.9 [54.7, 63.1]	57.5 [53.3, 61.7]	53.8 [50.0, 57.6]	62.9 [55.3, 70.5]	67.1 [60.6, 73.6]

^a^Values do not always add up to 100% because participants are weighted for representativeness.

^b^: Educational level according to ISCED but without vocational qualifications (not assessed)

### Feature evaluation and acceptance of scorings

Whereas the clear majority (72%) of our respondents evaluated the observation and scoring of texting while driving as justified (Table 2), situational features, driving during the day or at night and driving in rural or urban areas were deemed justifiable by only a minority (10% and 22%, respectively). For health insurance, the majority of our sample evaluated cancer screening participation (55%) and smoking status (58%) as justified features, while walking (18%) and sleeping (9%) were considered justifiable by only small minorities. Factors that influence justifiability were published in a report by the Advisory Council for Consumer Affairs Germany [45].

The acceptance of scoring systems was associated with the evaluations of different features in terms of their justified use for scoring. Justified use of all features increased (with odds ratios between 1.63 and 3.57) the chance of considering driver scoring. Based on justifiability ratings, a logistic regression explained 32% of driver scoring program acceptance (*χ*^2^(5) = 312.50, *p* < .001). Justified use of all features except sleep duration (*p* = .455) increased (with odds ratios between 1.75 and 2.71) the chance of considering health scoring. Based on justifiability ratings, a logistic regression explained 37% of variance in health scoring acceptance (*χ*^2^(6) = 323.27, *p* < .001).

**Fig 1:**
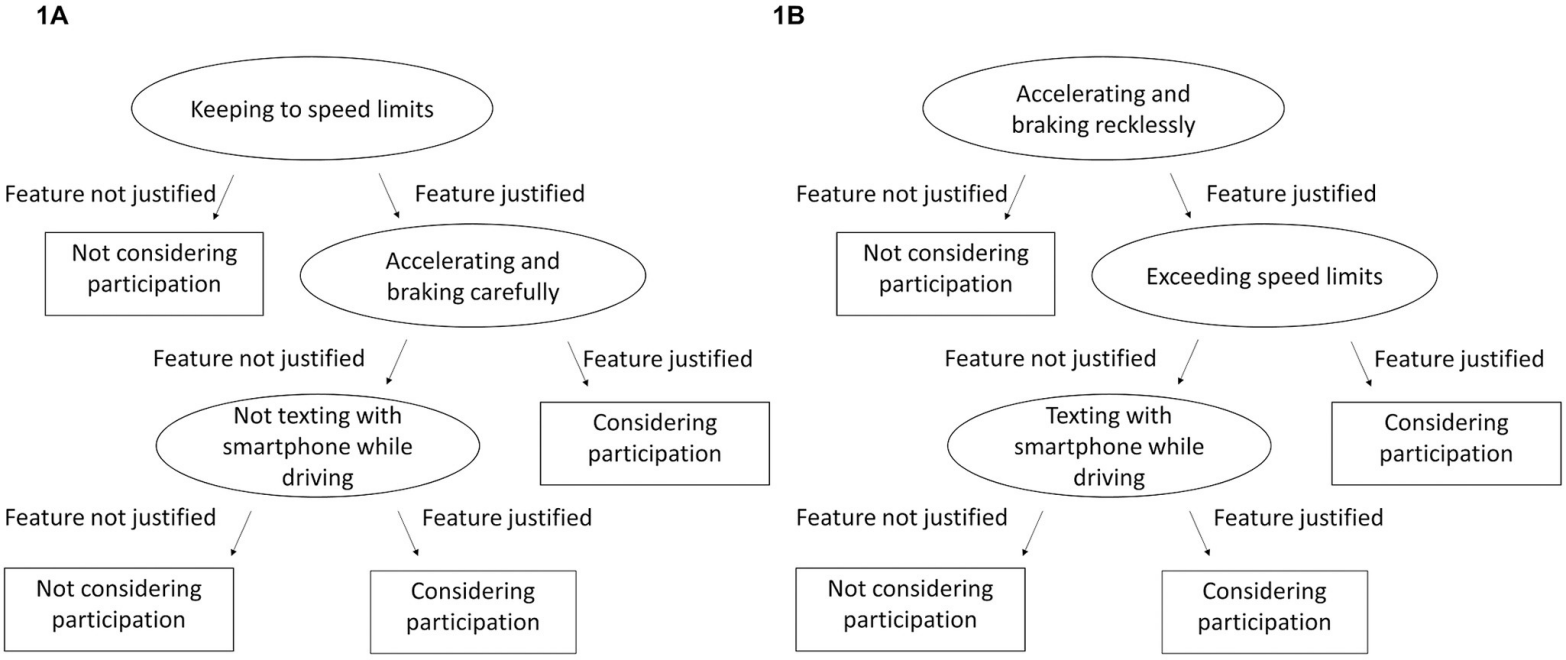
Fast-and-frugal decision trees (FFTs) for driver scoring with bonus (A) and malus framing (B).

In a next step, we used the evaluation of features to model participants’ decision process for considering or not considering health or driver scoring. Given the many factors that influence such a consideration, this task meets the definition of a problem under uncertainty [47]. Because simple models are useful for making predictions under uncertainty [48], we modelled the acceptance of scoring systems using fast-and-frugal decision trees (FFTs) [28]. These were shown to perform comparably well to highly complex models while being comprehensible (e.g. Fig 1A). Because it was reported before that framing affects the evaluation of individual scoring features [4], the respective uptake considerations were modelled separately.

Each FFT contains three features whose justifiability for driver scoring had been rated by participants (Fig 1A and 1B). According to the FFT for the bonus condition, if participants did not find it justified to reward adherence to speed limits, they did not consider participation in driver scoring. If participants found it justified to reward adherence to speed limits, careful acceleration and braking, they considered participating in driver scoring. Those who were against rewarding careful acceleration and braking considered participation if at least non-texting while driving was rewarded.

For the malus condition: If participants did not find it justified to punish reckless acceleration and braking, they did not consider participation in driver scoring. If participants found it justified to punish exceeding of speed limits and reckless acceleration and braking, they considered participating in driver scoring. Those who were against punishing violation of speed limits considered participation if at least texting while driving was punished.

Situational features were not predictive in either model of participants’ decision making. The trees’ predictive accuracy (balanced across misses and false positives) of.76 (for bonus) and.74 (for malus) was confirmed with 10fold cross-validations (training and testing data were randomly chosen ten times).

**Fig 2:**
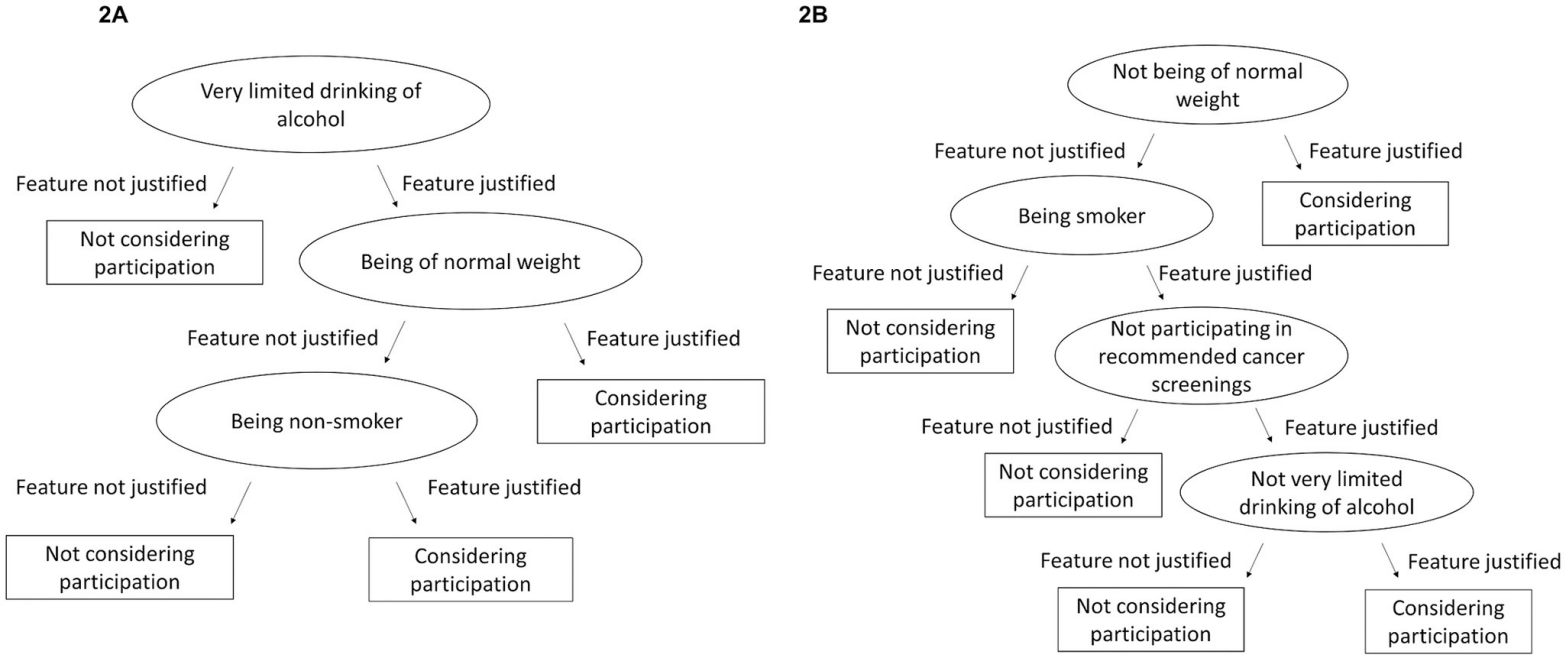
Fast-and-frugal decision trees (FFTs) for health scoring with bonus (A) and malus framing (B).

Based on the rated justifiability of feature use for health scoring, FFTs with three (bonus, Fig 2A) and four features (malus, Fig 2B) were modelled. According to the FFT for the bonus condition, if participants did not find it justified to reward limited alcohol consumption, they did not consider participation in health scoring. If participants found normal weight a feature worthy of being rewarded, they considered participating in health scoring. Those who were against rewarding limited alcohol consumption and body weight considered participation if at least non-smoking was rewarded.

For the malus condition, if participants found it justified to punish those with non-normal body weight, they considered participation in health scoring. If participants did not find it justified to punish non-normal body weight or smoking, they did not consider participation in health scoring. Only those in favour of punishing smoking, non-participation in cancer screening and high alcohol consumption considered participation.

Sleeping and walking evaluations did not predict health scoring considerations. Participation in cancer screening was relevant only for the bonus frame. The tree’s predictive accuracy of.75 (for bonus) and.74 (for malus) was confirmed with 10fold cross-validations.

### Are the features car and health insurers use valid and considered justifiable by consumers?

In the last step, participants’ evaluations of the justifiability of features to be used for scoring was contrasted with the actual use of features by insurers to calculate premium-related reward or punishment in driver and health scoring tariffs. First, we re-analysed company self-report data based on a survey which we designed and whose results were previously published [4]. Besides braking and acceleration, the feature speed was used by nearly all insurers (Table 1). Time of driving time and area were used by about one third of insurers. However, only two out of ten scored smartphone use in 2018. This is noteworthy because the justifiability of the feature, specifically, texting while driving, is relevant for predicting driver scoring acceptance (Fig 1A and 1B).

Screening participation and examinations, health course participation, sports club membership, sports awards and badges but also vaccination status were rewarded by nearly all health scoring programmes. Only 16–18% of programmes scored smoking and weight (BMI). This contrasts with the relevance of those features’ evaluation for the acceptance of health scoring (Fig 2A and 2B). Only 3 out of 45 insurers rewarded use of health apps and wearables in 2018.

Second, we collected available evidence on the validity of the features (Table 1), the effectiveness of features as regards the goal of driving safety and good health. “Valid” was assigned to general knowledge (e.g. speedometers can assess speed reliably, speed of driving is regularly modifiable without heavily affecting life conditions) and to evidence based on empirical trials, systematic reviews and consensus statements of research associations that we uncovered with systematic literature searches. “Unknown” was assigned to any other evidence (e.g. lack of or conflicting evidence). For driver scoring, only one feature could not be unambiguously confirmed by the literature, as the risk of driving in urban vs. rural areas depends on the type of safety event considered [32, 33].

For health scoring, in contrast, only half of the features could be confirmed to promote health. Regularly sleeping more than 8 hours is not necessarily healthy [38, 39]. Cancer screenings vary in their benefit-harm ratios, with no proof of all-cause mortality reduction for any type of cancer [42]. The thresholds of normal weight, the use of BMI and the individual level for health promotion are subject to conflicting evidence [41]. The unknown evidence status of normal weight contrasts with its perceived relevance for the acceptance of health scoring (Fig 2A and 2B). However, the evaluation of smoking and alcohol as determinants of health scoring acceptance is clearly in line with the clinical evidence.

To summarize, insurers in Germany rarely use the features which the participants consider to be the most relevant and justifiable, that is, smoking status for health scoring and smartphone use for driver scoring.

## Discussion

About every third person from 16 years of age in Germany—irrespective of gender—considers participation in driver scoring or health scoring (Table 2) that incentivises behaviour in line with the goals of the respective insurers. Younger people are overrepresented in this substantial minority; they pay higher premiums on car insurance than middle-aged people [49]. Moreover, young people show higher interest in consumer services that are realised with the help of information and communication technologies (telematics) and signal commitment to behavioural change [50]. Unlike in the case of driver scoring (bonus over malus), the population accepts health scorings with bonus and malus framing similarly.

Laypeople’s evaluation of selected scoring features may play a central role in their evaluation of a programme (shown by fast-and-frugal tree models). This provides insights into what likely drives people’s decisions whether to participate in the scoring programmes.

The first insight is the gap between the features accepted by consumers and the features used by insurers. Smartphone use (texting) while driving is a feature supported by a clear majority of the population (72%). It is also the only feature supported even more strongly when punished by malus [4]. This acceptance is in line with traffic risk evidence [51] and could be related to incidental news on accidents caused by smartphone-related distracted driving. The rare use of this feature in driver scorings in Germany (2018) clearly stands in contrast to evidence as well as to the lay evaluation. In accordance with that finding, our sample shows that more than half of the people in Germany support the evidence-based feature smoking for health scoring, but insurers rarely use it. One possible motive for not including texting while driving as a feature could be a concern with discouraging potential customers, given that more and more people text while drive. Yet the participants of the present study appear to accept such regulation by scoring. To that end, not only safety potential but also uptake potential is wasted. Including evidence-based and highly accepted features could support informed participation decisions.

The second insight is that feature acceptance seems to depend on fairness evaluations. Scoring situational features—time of driving (night or day) and area of driving (urban or rural areas)—is deemed least justified (10% and 22%). People may perceive their use as unfair. Under many conditions, people cannot simply choose where and when they drive: Health care employees working the night shift at emergency departments would be punished for night-time driving (the same for parents picking up their teenage children from parties at night). The practice of including features that are statistically associated with accidents but not under the customer’s control counteracts the stated goal of improving driving safety [52]. Although the programmes are not intended to produce fairer pricing of coverage, they likely aim at increasing the number of customers participating. Even algorithms that are transparent about feature weights enable insurers more control about individual premiums, e.g. they can modify algorithms and how much they reward or punish quickly. Yet customer participation is likely to be driven by fairness concerns [53], as this study indicates.

A central research question is posed by the link between the acceptance of features and their evidence basis, because there are scoring features that are currently used, which are proxies (e.g. external validity of gym memberships) or hardly evidence-based (e.g. internal validity of skin cancer screening [42]). The present study does not enable us to determine the degree to which the limited validity of some of the features used by insurers hinders the uptake of telematics tariffs and incentive programmes of health insurers (only 3 out of 45 insurers score more than 20% of their customers [4]). We hypothesise that it is the validity of perceived features rather than actual knowledge, which influences programme acceptance. For instance, the population strongly overestimates the benefits of cancer screenings [54], a widely implemented feature in health scoring, for which our study shows high acceptance rates.

To this end, in order to increase acceptance insurers might consider (i) basing scoring systems on features that do not discriminate against people who have no possibility to avoid certain conditions (e.g. driving at night), and (ii) communicating the validity of the features, that is, the scientific evidence. This also calls for randomised-controlled studies proving effectiveness of scoring programmes in terms of the announced goals: driving safety, operationalised by accidents and their consequences, and health, operationalised by clinical outcomes. Political stakeholders can incentivise insurers to generate and communicate this evidence for the benefit of public safety.

The key limitation of our survey study is that we have investigated behavioural intentions only, not actual behaviour. The scoring scenarios we used were short, as necessitated by telephone surveys, and could have neglected relevant real-world features. Given 10% response rate, though usual for random digit calls to landline and mobile, a response bias could have evoked when the Ministry of Justice and Consumer Protection or the topics of health, car and data protection were mentioned in recruitment. We have to assume that more critical citizens, who more likely reject consumer scoring programmes or certain features, could not be sampled representatively. Accordingly, the absolute results for the population in Germany could be even more critical, with less proponents of such programmes. Finally, it would be desirable to cross-validate the FFT models in other cultures, as well as with a sample that receives more detailed information about scoring programmes.

Finally, this paper does not explore privacy concerns about device-based telematic solutions (e.g. in mobile Health [55]). These remain relevant even if the proposed measures of fairness and evidence-based information are implemented in scoring programmes [56].

To sum up, future research can be based on our insights that laypeople need reliable information about why certain features can be used to affect pricing of car and health insurance programmes. Reliable information plays an important role for their acceptance of such scorings. However, at the moment, justifiable features, from the perspective of potential customers, are rarely used.

## Supporting information

S1 Table Introduction and consent form for the random digit call. (DOCX)

S2 Table Items in the questionnaire. (DOCX)

S3 Table Supplementary information on the regression analyses. (DOCX)
