# Supplementary material for: Acceptance of criteria for health and driver scoring in the general public in Germany
Source: PLoS One. 2021 Apr 22;16(4):e0250224. doi: 10.1371/journal.pone.0250224 (PMC8062065; doi:10.1371/journal.pone.0250224)
Supplement: S1 Table — (DOCX) [file pone.0250224.s001.docx]

**S1 Table. Introduction and consent form for the random digit call**

| **Text** |
| --- |
| Hello, my name is ........... from the infas Institute in Bonn! |
| We are conducting a survey on behalf of the Federal Ministry of Justice and Consumer Protection on various current topics relevant to consumers, for example in the areas of health, cars and data protection. |
| FURTHER INFORMATION IS AVAILABLE ON REQUEST: |
| - The interview lasts about 20 minutes. |
| - The survey is conducted for the Expert Council on Consumer Affairs. This is an advisory body for the Federal Ministry of Justice and Consumer Protection (BMJV). With your participation you can make a valuable contribution to consumer protection in Germany. |
| - Your household was selected by a random sample. |
| - The results of the survey will be analysed exclusively anonymously and separately from your telephone number. |
| - infas works strictly according to the legal provisions of data protection. |
| - Participation is of course voluntary. |
| IF landline |
| - For this purpose, I would like to speak to the person in your household who is at least 16 years old and had their last birthday. |
|  |
| IF mobile phone |
| - For this I would like to speak to the main user of the mobile phone on which we have now reached you who is at least 16 years old. |
| - INT: If the target is not on the line, please ask for the target person and read out the introductory text again. Clarify that the primary user is at least 16 years old. |
| (all) |
| Can we start the interview now? |
| DOCUMENTATION  I1=1 Interview  I1=2 Appointment  I1=3 hangs up immediately  I1=4 refuses  if I1=2: appointment  RCL 6: vague appointment  RCL 7: definite appointment  RCL 47: interview interrupted: will be continued -> note appointment  if I1=4: refused  RCL 16: target group not reached, no one with at least 16 in HH/handy user under 16  RCL 17: ZP already interviewed  RCL 13: no private person  RCL 42: not available in field time  RCL 43: respondent not able to be interviewed/ permanently ill or disabled  RCL 52: access to CP prevented > PLEASE EXPLAIN  RCL 53: CP refuses to provide any information  RCL 54: Refusal: not interested/because of the issue  RCL 9: Refusal: no time  RCL 54: refusal: too many surveys  RCL 59: refusal: data protection reasons  RCL 9: refusal: length of interview  RCL 11: refusal: ZP ill  RCL 8: refused in principle  RCL 62: ZP refused: other reasons > PLEASE EXPLAIN  RCL 80: insufficient knowledge of German  RCL : does not wish to participate by telephone |
| Nice of you to join us. Then we will start the interview now. |
