# Supplementary material for: Acceptance of criteria for health and driver scoring in the general public in Germany
Source: PLoS One. 2021 Apr 22;16(4):e0250224. doi: 10.1371/journal.pone.0250224 (PMC8062065; doi:10.1371/journal.pone.0250224)
Supplement: S2 Table — (DOCX) [file pone.0250224.s002.docx]

**S2 Table. Items in the questionnaire**

| Scoring | Bonus framing | Malus framing |
| --- | --- | --- |
| Driver scoring | Imagine that a car insurance company offers a tariff that depends on the driver's driving behaviour. The driving behaviour would be recorded for this purpose, for example with a cell phone. Whoever participates in this tariff could, depending on their own driving behaviour, influence the amount of their own insurance premium. How would you evaluate the following regulations? | |
| Items | Lower car insurance premium is paid by those who...  ...maintain the prescribed maximum speed  ...do not write or read cell phone messages while driving  ...mostly drive during the day  ...mostly drive a car in the country  ...accelerate or brake carefully | Higher car insurance premium is paid by those who...  ...exceed the prescribed maximum speed  ...write or reads cell phone messages while driving  ...mostly drive at night  ...mostly drive a car in the city  ...carelessly accelerate or brake |
|  | Fully justified  Rather justified  Rather unjustified  Not justified at all  Refused/white no | |
|  | Would you personally use such a car insurance tariff that takes into account features such as speed, mobile phone use, acceleration and braking behaviour, time and area of driving? | |
|  | Yes  No  I do not know | |
| Health scoring | Imagine that a health insurance company offers a tariff that depends on the health behaviour of the insured. The health behaviour would be recorded for this purpose, for example using a cell phone. Who participates in this tariff, could, depending on the own health behaviour, influence the amount of their own insurance premium. How would you evaluate the following regulations? | |
| Items | Lower car insurance premium is paid by those who...  ...walk at least 6 kilometres per day  ...sleep at least 7 to 8 hours per night  ...drink only small amounts of alcohol  ...are of normal weight  ...participate in recommended cancer screening tests  ...are non-smokers | Higher car insurance premium is paid by those who...  ...walk less than 6 kilometres per day  ...sleep less than 7 to 8 hours per night  ...drink more than small amounts of alcohol  ...are overweight  ...do not participate in recommended cancer screening tests  ...are smokers |
|  | Is fully justified  Rather justified  Rather unjustified  Not justified at all  Refused/white not | |
|  | Would you personally use such a health insurance tariff which takes into account features such as step count, sleep, alcohol consumption, smoking and body weight? | |
|  | Yes  No  I do not know | |
