# Supplementary material for: Acceptance of criteria for health and driver scoring in the general public in Germany
Source: PLoS One. 2021 Apr 22;16(4):e0250224. doi: 10.1371/journal.pone.0250224 (PMC8062065; doi:10.1371/journal.pone.0250224)
Supplement: S3 Table — (DOCX) [file pone.0250224.s003.docx]

**S3 Table. Supplementary information on the regression analyses.**

| **Result section** | **Analysis** | **Variables included in the regression; coefficients with standard error** | **p** | **OR [95%CI]** | **Goodness of fit** |
| --- | --- | --- | --- | --- | --- |
| Acceptance of driver scoring, health scoring, and respective scoring features | Intention to participate in driver scoring | Age: -0.46 (0.13) Gender: -  Education: - | <.001  .127  .172 | 0.63 [0.38, 0.88]  -  - | χ^2^(4) = 41.82 |
|  | Intention to participate in health scoring | Age, gender, education: - | - | - | χ^2^(4) = 1.84 |
| Framing effect of bonus and malus | Participating in driver scoring | Malus frame: -0.57 (0.12) | <.001 | 0.57 [0.31, 0.81] | χ^2^(1) = 21.16 |
|  | Participating in health scoring | Malus frame: - | - | - | χ^2^(1) = 1.80 |
|  | Speed limit violation justifiability | Malus frame: -1.36 (0.13) | <.001 | 0.26 [0.01, 0.51] | χ^2^(4) = 144.15 |
|  | Night time driving justifiability | Malus frame: -1.87 (0.26) | <.001 | 0.16 [0.00, 0.67] | χ^2^(4) = 80.40 |
|  | Urban driving justifiability | Malus frame: -0.92 (0.15) | <.001 | 0.40 [0.11, 0.69] | χ^2^(4) = 52.66 |
|  | Texting while driving justifiability | Malus frame: 0.67 (0.14) | <.001 | 1.95 [1.69, 2.21] | χ^2^(4) = 36.06 |
|  | Accelerating and braking justifiability | Malus frame: - | .203 | - | - |
|  | Too little walking justifiability | Malus frame: -1.36 (0.19) | <.001 | 0.26 [0.00, 0.63] | χ^2^(4) = 69.86 |
|  | Little sleep justifiability | Malus frame: -1.08 (0.25) | <.001 | 0.34 [0.00, 0.83] | χ^2^(4) = 28.31 |
|  | Too much weight | Malus frame: - | .058 | - | - |
|  | Too much alcohol justifiability | Malus frame: -0.33 (0.13) | .011 | 0.72 [0.47, 0.97] | χ^2^(4) = 10.89 |
|  | Non-participation in cancer screening justifiability | Malus frame: -0.64 (0.13) | <.001 | 0.53 [0.28, 0.78] | χ^2^(4) = 40.17 |
|  | Smoking justifiability | Malus frame | .781 | - | - |
| Modelling participants’ acceptance of health and driver scoring | Intention to participate in driver scoring | Speed  Texting  Driving time  Driving area  Accelerating and braking | <.001  <.001  .042  .004  <.001 | 3.57 [3.28, 3.86]  2.10 [1.73, 2.47]  1.64 [1.16, 2.12]  1.63 [1.30, 1.96]  3.34 [3.06, 3.63] | χ^2^(5) = 312.50 |
|  | Intention to participate in health scoring | Walking  Sleeping  Alcohol  Weight  Cancer screening  Smoking status | <.001  .455  <.001  .001  <.001  <.001 | 2.67 [2.26, 3.07]  -  2.71 [2.38, 3.03]  2.79 [2.45, 3.12]  1.75 [1.41, 2.09]  2.17 [1.79, 2.55] | (χ^2^(6) = 323.27 |
